# The significance of nonurgent psychiatric emergencies in an ED: a retrospective study

**DOI:** 10.1186/s12873-023-00900-z

**Published:** 2023-11-08

**Authors:** Heribert Kirchner, Heiko Ullrich, Peter Neu, Nik Hulsmans, Georg Juckel, Patrick Brzoska

**Affiliations:** 1 Faculty of Health, School of Medicine, University Witten/Herdecke, Herrhausen-Straße 50, 58455 Witten, Germany; 2 Center of Mental Health, Kreisklinikum Siegen, Weidenauer Str. 76, 57076 Siegen, Germany; 3 Department of Psychiatry and Psychotherapy, Jewish Hospital Berlin, Heinz-Galinski-Straße 1, 13347 Berlin, Germany; 4 Department of Psychology, University of Siegen, Adolf-Reichwein-Straße 2a, 57076 Siegen, Germany; 5 Department of Psychiatry, Psychotherapy and Preventive Medicine, LWL University Hospital, Ruhr University Bochum, Alexandrinenstraße 1, 44791 Bochum, Germany

**Keywords:** Emergency, Psychiatric, Emergency room, Hospital, Nonurgent, Mental health conditions

## Abstract

**Background:**

In emergency departments, patients with mental health conditions are a major concern and make up the third or fourth of the most common diagnosis seen during all consultations. Over the past two decades, there has been a noticeable rise in the number of cases, particularly due to an increase in nonurgent visits for somatic medical issues. The significance of nonurgent visits for psychiatric patients is yet to be determined. This study aims to uncover the significance and identify the characteristics of this group.

**Methods:**

A retrospective analysis of psychiatric emergency visits at an interdisciplinary emergency department of a German general hospital in 2015 was conducted. For this purpose, patient records were reviewed and evaluated. An analysis was conducted based on the German definition of psychiatric emergencies according to the German guidelines for emergency psychiatry.

**Results:**

A total of 21,124 emergency patients visited the evaluated Emergency Department. Of this number, 1,735 psychiatric patient records were evaluated, representing 8.21% of the total population. Nearly 30% of these patients did not meet any emergency criteria according to German guidelines. Significant differences were observed between previously treated patients and those presenting for the first time.

**Conclusions:**

The high proportion of nonurgent psychiatric patients in the total volume of psychiatric emergency contacts indicates a possible control and information deficit within the emergency system. Just as prior research has emphasized the importance of investigating nonurgent somatic medical visits, it is equally imperative to delve into studies centered around psychiatric nonurgent presentations.

## Background

Global research efforts highlighted the relevance of PE (PE) patients concerning the level and consumption of resources in emergency departments (EDs). Based upon recent study results, initial conclusions can be drawn regarding underlying causes for presentation, irrespective of the nation and health care system involved [1–4]. In Germany, approximately 9.8 million outpatients received emergency treatment in 2021 in EDs [5]. Psychiatric emergencies account for approximately 5–10% of this population [1]. The proportion of nonurgent PE patients among this large number of ED patients cannot be determined solely based upon previous literature, mainly since research on this niche issue remains scarce. Furthermore, individuals who experience physical symptoms that are actually due to a mental health condition pose an additional difficulty in the emergency department. For example the significance of mental health conditions in older multimorbid patients presenting to the emergency department for acute cardiac symptoms is described by Figura et al. in 2021 [6]. However, it is important to determine whether nonurgent psychiatric patients could also contribute to increasing caseloads presenting at EDs, especially considering the growing number of patients presenting to EDs globally [4], linked with possible negative implications [4, 7]. For instance, a higher risk for decreased treatment quality due to overcrowding scenarios can be a possible consequence [3, 8]. Given this context, scientific interest in this issue has increased significantly [9]. To the best of the authors' knowledge, however, no studies have been conducted that characterize patients and their urgency, based upon individual psychiatric conditions, for presentation. Such a niche research field remains in scarcity, with only two USA epidemiological studies being particularly noteworthy [1, 2]. In both studies, an increase in the number of cases and the range of diagnoses (particularly substance-related presentations, as well as affective disorders and anxiety panic disorders) were reported in EDs. In Europe, studies on the range of diagnoses and their frequency were conducted [10, 11], through there was no examination of nonurgent psychiatric patients within EDs, that are typically available to patients 24/7 and serve only as the point of contact for all medical emergencies in a hospital [7]. Consequently, insights into psychiatric urgency and its proportion among total PEs within EDs cannot be performed, as based on current data. There have been efforts in the past two decades, in countries such as Australia and the USA, to implement and study specific triage systems for psychiatric patients in EDs [12–14]. The goal was to provide appropriate care for this group of patients, based on their level of urgency, similar to management protocols for somatic medical emergencies, using specific criteria [12–14]. Additionally, the development of specific indicators, such as urgency, patient characteristics, and patient populations, is becoming increasingly important for future health policy decisions regarding demand-driven care, including for the psychiatric patient group [15, 16]. In this context, the first mental health surveillance projects emerged in Europe and USA [10, 15, 17, 18]. In Germany, researchers at the Robert Koch Institute are currently developing a tool to track utilization patterns of PE patients in EDs on a weekly basis, with initial results being highly encouraging [10].

Conversely, research on low-urgency somato-medical emergency contacts is well-documented [19–21]. Regardless of which ED was investigated, the reasons for nonurgent somato-medical patient visits were very similar throughout [8, 9]. Patients sought ED treatment due to their subjectively perceived exceptional health situation. The main reasons included long waiting times for appointments with specialists, together with low-threshold / competent 24/7 services offered by the ED. Interestingly, a significant proportion of nonurgent patients still considered themselves to require emergency treatment [9, 10]. Frequently changing, less stable relationships with a primary care physician were also cited as a contributing factor for patients to attend the ED [11].

It is important to recognise that the General Practitioner (GP) plays an important role within the German healthcare system. The German outpatient healthcare system is an integral part of the country's comprehensive healthcare system. The outpatient sector refers to medical care and services provided to patients who do not require hospitalization. It plays a crucial role in delivering primary and specialized healthcare to the population. The cornerstone of the German outpatient system is the role of the GP or ‘Hausarzt’. Patients in Germany typically register with a specific GP, who serves as their primary care provider. These doctors are often the first point of contact for patients seeking medical care and are responsible for coordinating and managing their healthcare needs. While GPs provide primary care, patients can also directly access specialists, such as psychiatrists, neurologists or cardiologists, without requiring a GP referral. This system allows patients to seek specialized care when necessary, though many still prefer to consult their GP first for a referral. Patients can also present themselves directly to an ED in case urgent healthcare requirements. The EDs are often located at the local hospital. Various reasons can be found for the increased use of EDs. For example, the lack of GPs among younger, generally healthy patients is one of the causes of nonurgent ED use. Moreover, the age-related declining number of GPs and specialists in rural areas leads patients to the emergency department. This is a result of the lack of timely resources in practices [22]. Due to long waiting times for GP / specialist appointments, patients attempt to bypass normal protocols and present at the ED without consulting their GP beforehand. This phenomenon has occurred frequently during the last decade. The shortage of psychiatrists is also a significant issue in Germany.

Within the investigated city of Siegen, outpatient psychiatric and neurological care is currently provided by seven psychiatric and four neurological practices, in addition to a psychiatric outpatient clinic at the hospital. Currently, in Germany, mean waiting time for initial specialist care is a minimum of approximately four months in urban areas and approximately 5–6 months in rural areas [23]. The aim of this study was to determine the share of nonurgent patients among the total number of PE visits.

## Methods

This was a retrospective study of all PE cases at the interdisciplinary ED of a hospital in Siegen, Germany, utilizing data across 2015. A secondary data analysis – that incorporated a cross-sectional design—was selected. All collected hospital-derived data were sent to the study group anonymously. An existing and already published dataset was subjected to a re-analysis regarding the relevance of nonurgent psychiatric emergency patients [24].

The study was approved by the Scientific and Research Ethics Committee of the Medical Research Council of the University of Münster, Germany (No. 2019–529-f-S, Ethik-Kommission/Ärztekammer Westfalen-Lippe, Germany) and carried out in accordance with the tenets of the Declaration of Helsinki. The need for consent to participate was waived by an Institutional Review Board (IRB/Ethik-Kommission/Ärztekammer Westfalen-Lippe, Germany).

In 2020, a specialist in psychiatry and psychotherapy at the hospital collected data from all adult patients presenting to the ED with a final primary psychiatric diagnosis (18 years and older). Pre-training was conducted on examples of urgent and nonurgent PEs to improve reliability (according to the definition of the 2019 DGPPN S2k guidelines for emergency psychiatry).

Patients who did not fulfil the criteria for absolute and/or relative urgency were considered to be nonurgent psychiatric patient contacts and were included in the study. Table 1 lists all PE syndromes and is based on the 2019 DGPPN S2k guidelines for emergency psychiatry.

**Table 1:** Psychiatric emergency criteria (German Guideline – DGGPN 2019)

Absolute Psychiatric Emergency	Relative Psychiatric Emergency
- Suicide attempt	- Confusion
- Concrete suicide ideas/plans	- Withdrawal without delirium
- Severe intoxication	- Suicidality without intention
- Severe state of arousal	- Anxiety and panic disorder
- Aggressiveness/violence caused by mental disorder	- Acute adjustment reaction and psychosocial dysfunction
- Delirium	

List of all important absolute and relative psychiatric emergencies according to the German guideline for emergency psychiatry (2019)

Legend: The concept of psychiatric emergencies is further subdivided into absolute and relative emergencies. Syndromes associated with absolute psychiatric emergencies differ in severity and demand immediate action, whereas relative psychiatric emergencies do not have the same level of urgency

The first step involved assigning a syndrome classification to each documented clinical scenario, based upon absolute and/or relative emergency criteria, using individual evaluations of patient records. Subsequently, nonurgent patients were grouped according to their respective associated primary diagnoses, as defined by the International Classification of Diseases 10th Revision (ICD-10). For example, all intoxications and withdrawal syndromes were categorized under F1 (Mental and behavioural disorders due to psychoactive substance use). This classification was applied to all psychiatric primary diagnoses, ranging from F0-F7 or X-coding for suicidal tendencies. In addition, the following factors were included in the analysis: age, gender, day of the week, time of day, month, previous outpatient or inpatient psychiatric treatment at the investigated hospital, admission to hospital due to legal measures, referral by a physician, route of access (emergency medical and ambulance service, police, self-initiated), emergency psychiatric syndrome, existence of suicidal tendencies.

The unique feature of this ED is that nearly all PEs of the investigated city- at least during this timeframe—were brought to the hospital by the rescue service. Additionally, a significant number of individuals seeking psychiatric care also visited the ED as self-presenters. Consequently, this ED plays a crucial role in providing PE services in Siegen. At the time of the study, the hospital had 556 beds, including 11 specialty clinics and a psychiatric department with 140 beds. It is responsible for providing medical care to the entire district, which has a population of 280,000. The focus of this study was to identify cases of nonurgent psychiatric patient contacts.

The statistical analysis was performed using SPSS® v.25. The comparison between independent groups (urgent vs. nonurgent emergency contact) for continuous variables was conducted using either an independent-samples T-test (when normal distribution was assumed) or a Mann–Whitney U test (when normal distribution was not assumed). To compare the frequency distribution of categorical variables between independent groups, the chi-square test (all other variables when cell frequencies were > 5) or Fisher's exact test (ICD-10: F5, F7, antipsychotic medication, antidepressant, internal medication, other medication) or Monte Carlo simulation (age and mode of transportation, when cell frequencies were < 5) was used. Unless otherwise specified, a significance level of *P* < 0.05 was used to determine statistical significance. All tests were two-tailed, and the analysis was purely exploratory, so p values were interpreted descriptively.

## Results

In 2015, 21,124 emergency patients visited the interdisciplinary ED of the examined hospital. Among these, psychiatric patient records were identified in a total of 1735 cases, which accounted for 8.2% of the total number of emergency patients.

Out of the 1735 psychiatric cases, 512 did not meet the criteria for absolute and/or relative emergency status (see Table 2), constituting 29.5% of the psychiatric patient volume for the entire year.

**Table 2:** Frequencies and group comparison of categorical variables

Variable		Non urgent (*n* = 512)		Urgent (*n* = 1177)		*p*^*a*^
		Frequency	Percentage	Frequency	Percentage	
Sex	Female	244	47.7	572	48.6	.751
	Male	268	52.3	605	51.4	
Age	< 18	2	0.4	6	0.5	.105^d^
	18–29	133	26.0	299	25.4	
	30–39	126	24.6	257	21.8	
	40–49	105	20.5	205	17.4	
	50–59	81	15.8	202	17.2	
	60–69	34	6.6	86	7.3	
	70–79	22	4.3	72	6.1	
	80–89	9	1.8	44	3.7	
	90–99	0	0	6	0.5	
Known patient	Yes	235	45.9	626	53.2	.007
	No	276	53.9	551	46.8	
	Missing	1	0.2	0	0	
Time	00:00–03:59	33	6.4	101	8.6	< .001^b^
	04:00–07:59	13	2.5	55	4.7	
	08:00–11:59	148	28.9	219	18.6	
	12:00–15:59	145	28.3	306	26.0	
	16:00–19:59	101	19.7	256	21.8	
	20:00–23:59	72	14.1	240	20.4	
Legal Status	Voluntary	507	99.0	951	80.8	< .001^b^
	Involuntary hospitalization (German PsychKG)	2	0.4	136	11.6	
	Involuntary hospitalization (German BtG)	3	0.6	89	7.6	
	Missing	0	0	1	0.1	
Admission	Yes	173	33.8	855	72.6	< .001
	No	339	66.2	332	27.4	
	Missing	-	-	-	-	
Referral to Hospital	Yes (by GP)	34	6.6	126	10.7	< .001^b^
	Yes (by Emergency Physician)	8	1.6	72	6.1	
	No	467	91.2	971	82.5	
	Missing	3	0.6	8	0.7	
Mode of Transportation	By Foot	451	88.1	573	48.7	< .001^d^
	Paramedics	55	10.7	502	42.7	
	Police	3	0.6	92	7.8	
	Others	2	0.4	1	0.1	
	Missing	1	0.2	9	0.8	
Main Diagnosis F0 Organic, including symptomatic, mental disorders	Yes	8	1.6	78	6.6	< .001
	No	504	98.4	1099	93.4	
	Missing	-	-	-	-	
Main Diagnosis F1 Mental and behavioural disorders due to psychoactive substance use	Yes	142	27.7	363	30.8	.204
	No	370	72.3	814	69.2	
	Missing	-	-	-	-	
Main Diagnosis F2 Schizophrenia, schizotypal and delusional disorders	Yes	61	11.9	272	23.1	< .001
	No	451	88.1	905	76.9	
	Missing	-	-	-	-	
Main Diagnosis F3 Mood [affective] disorders	Yes	139	27.1	215	18.3	< .001
	No	373	72.9	962	81.7	
	Missing	-	-	-	-	
Main Diagnosis F4 Neurotic, stress-related and somatoform disorders	Yes	148	28.9	208	17.7	< .001
	No	364	71.1	969	82.3	
	Missing	-	-	-	-	
Main Diagnosis F5 Behavioural syndromes associated with physiological disturbances and physical factors	Yes	3	0.6	3	0.3	.375^c^
	No	509	99.4	1174	99.6	
	Missing	-	-	-	-	
Main Diagnosis F6 Disorders of adult personality and behaviour	Yes	28	5.5	45	3.8	.151
	No	484	94.5	1132	96.2	
	Missing	-	-	-	-	
Main Diagnosis F7 Mental retardation	Yes	1	0.2	13	1.1	.077^c^
	No	511	99.8	1164	98.9	
	Missing	-	-	-	-	
Attempted suicide X84	Yes	0	0	21	1.8	.003
	No	512	100	1154	98.0	
	Missing	0	0	2	0.2	
Intoxication T36-T50	Yes	0	0	18	1.5	.008
	No	512	100	1156	98.2	
	Missing	0	0	3	0.3	

For 12 variables, there is a statistically significant difference between the two groups at a level of α < .05

^a^ For the calculation of the *p*-values, the cases of the category "Missing" were not considered

^b^ Asymptotic significance, since exact significance could not be calculated

^c^ Exact Fisher's test, since cell frequency < 5

^d^ Monte-Carlo simulation (10,000 samples), since cell frequency < 5 and no 2 × 2 matrix

The mean age of PE patients was 43.5 years (SD 17.8), while mean age of nonurgent psychiatric patients was 41.4 (SD 15.7) years, though no statistically significant variation was observed between such groups. The results demonstrated that previously established psychiatric patients were significantly more likely to fulfil the emergency criteria in comparison to first-time psychiatric patients.

**Fig. 1:**
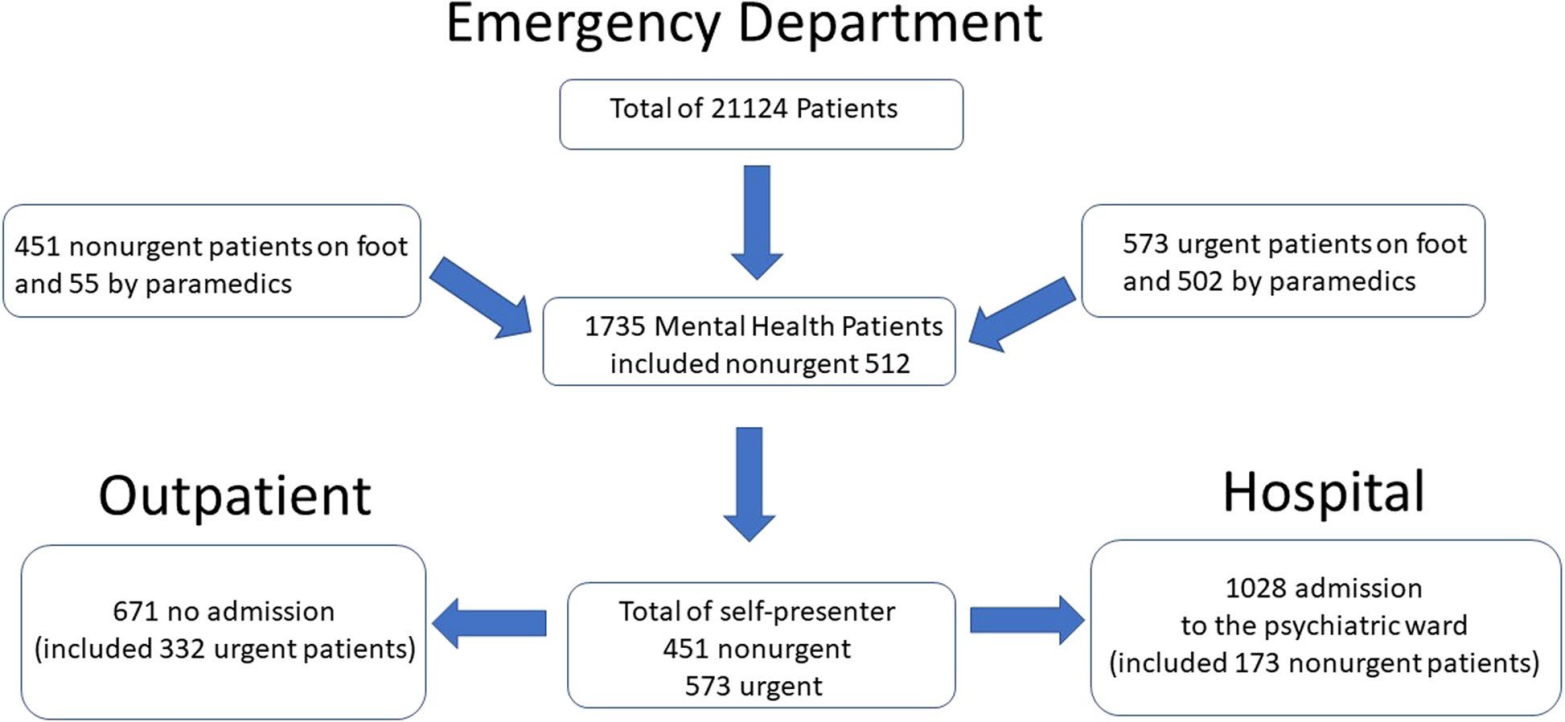
Emergency room patient flows. Legend: There are clear differences between the urgent and nonurgent group in terms of presentation mode and admission rate

Patients with nonurgent conditions visited the ED significantly more often during regular office hours (8 am—8 pm), and almost 80% of all nonurgent patients presented to the ED (*p* < 001). The rate of admission for nonurgent psychiatric patients into ED was 33.8%, which was significantly lower than the rate of almost 73% for patients with psychiatric emergencies (*p* < 001). Approximately 33% of nonurgent psychiatric patients were hospitalised (see Fig. 1).

**Fig. 2:**
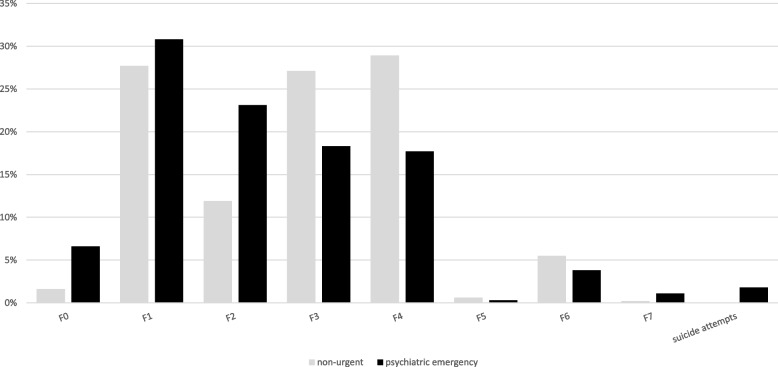
Main Diagnoses according to ICD-10. Legend: Variations across main diagnoses, subdivided into urgent and nonurgent mental health patients. Substance-related presentations play a major role in both urgent and nonurgent mental health cases

Only a minor segment of the nonurgent patient group (approximately 10%) sought treatment from a GP prior to presentation. Conversely, urgent PE patients were almost four-fold more likely to have been examined by a GP beforehand (*p* < 001). The majority (88.1%) of nonurgent psychiatric patients arrived at the ED as self-presenters, compared to only 48.7% for urgent patients (*p* < 001). Over 50% of patients were brought to the hospital with an ambulance. There were distinct differences in the distribution of diagnoses between the two groups. The urgent patient group had a higher proportion of F0 (organic, including symptomatic, mental disorders) and F2 (schizophrenia, schizotypal and delusional disorders) main diagnoses (*p* < 001), while the nonurgent patient group had a higher proportion of F3 (Mood (affective) disorders) and F4 (neurotic, stress-related and somatoform disorders) main diagnoses (*p* < 001). Significant variations were found in the distribution of main diagnoses between urgent and non-urgent psychiatric ED patients (see Fig. 2).

## Discussion

The aim of this study was to explore the proportion of nonurgent psychiatric patients in an ED at a German general hospital. This was the first investigation of its kind in Germany. The results of this study demonstrated that there was a possible underestimation of nonurgent psychiatric patient presentation frequency in the ED. In the studied cohorts, the majority (88.1%) of nonurgent psychiatric patients arrived at the ED as self-presenters, and there were distinct variations in the distribution of diagnoses between both groups.

Overall, patients with nonurgent treatment needs are becoming an increasing challenge for EDs in Germany [20, 21]. To date, existing studies have analysed changes in utilization behaviour among somato-medical patients [19–21], although a study of nonurgent psychiatric presentations and their proportion within total emergency care statistics in EDs has not been conducted until now. If there is frequent use of emergency resources by nonurgent psychiatric patients, this could lead to unnecessary resource allocation and negative consequences, such as increased risk of overcrowding and decreased quality of treatment. The results of this work, as described below, should serve as a foundation for mitigating potential overcrowding and resource allocation issues.

Within this study, psychiatric patients accounted for 8.2% of all patient visits to the ED, which is consistent with current data [1–3]. However, within the investigated cohorts, a high proportion of nonurgent psychiatric patient visits (approximately 33% of all psychiatric visits) was observed. Based on the definition of the German S2k-guideline (DGPPN) [15] on psychiatric emergencies, these patients did not meet the criteria, so they could have received treatment from a GP or specialist practice instead. Based on this study’s results, nonurgent psychiatric patient visits are relevant in the investigated ED, similar to the results found for nonurgent somatic patient visits [11, 15, 16]. In their 2020 study, Reinhold et al. found that EDs are facing an increase in case numbers and a high proportion of low urgency cases [25]. In their 2017 study, Schmiedhofer et al. found that a large proportion of patients presented to an interdisciplinary ED due to long waiting times in the outpatient setting and since such patients perceived the ED to have high levels of professional competence [19]. Other authors, such as Somasundaram et al. and Reins et al., agreed that patients' subjective assessment of urgency often differs greatly from professional assessment [20, 26]. To address this issue, several measurements were undertaken. In cooperation with the Association of Statutory-Health-Insurance, accredited physicians provide support, therefore patients can be distributed early between emergency outpatient departments and care within the Association of Statutory-Health-Insurance accredited physicians' system, thus relieving the emergency system. In addition, a central telephone number has been set up and advertised nationwide, which directs outpatients to the Association of Statutory-Health-Insurance accredited physicians' system if they do not know where to seek such medical advice. This is also intended to relieve EDs. The retrospective design of the study leaves the question on the patients' subjective assessment of urgency unanswered and should be explored in future studies. The high proportion of nonurgent psychiatric patients found in this study underlines the international scientific debate that began approximately two decades ago. Following such recommendations, health researchers placed effort to implement a modified triage system for psychiatric patients within ED settings [12, 13]. Initial modifications were recently developed in differing countries / triage systems (e.g., Manchester Triage System in the UK). The documentation logs for emergency staff were also modified. An overall need to develop and introduce nationwide ‘surveillance instruments’ to react quickly to changing trends, or to identify possible unwanted developments in EDs, was identified. The goal was to help in the decision-making process in terms of health policy. In several countries, several promising pilot projects already exist [10, 15, 16, 18, 27]. No significant differences were found between the nonurgent psychiatric patient and PE groups in terms of age and gender, which was in line with current data. The majority of nonurgent patients presented themselves ‘on foot’ during regular office hours and were first-time presenters at the ED, indicating that they could have sought alternative care due to long waiting times or immediate concerns. Only 10% of nonurgent patients had seen a GP prior to their visit to the ED. These findings support previous studies suggesting that the ED could be used as an alternative to office-based practices [21].

The rate of admission for nonurgent patients into the ED was 33.8%, which was significantly lower than the rate of nearly 73% for PE patients. Despite this, it was surprising that one-third of patients were admitted as inpatients, even though they did not meet the criteria for an emergency. Previous studies overall indicated an admission rate of approximately 50% for psychiatric emergencies in EDs, though these studies did not differentiate between nonurgent and absolute/relative PEs, which could justify variations in proportions observed in this study (33.8% and 73.0%, respectively). International research has identified various reasons for psychiatric inpatient admission, such as bed occupancy, lack of social networks, and homelessness. However, it was not possible to determine if these factors played a role in this study due to experimental design, and further research is necessary. The analysis showed clear differences between the two groups concerning underlying diagnoses, with the exception of substance-related presentations. Substance-related presentations were highly relevant in both groups. The nonurgent patient group exhibited high rates of affective disorders (main diagnosis ICD-10 F3) and neurotic, stress, and somatoform disorders (main diagnosis ICD-10 F4). These results suggest that several patients approach the ED with disorders that can be effectively managed within an outpatient setting. Studies identified a significant proportion of patients approaching EDs with anxiety / panic / minor affective disorders [28, 29]. In conclusion, the results highlight that a relevant proportion of patients who visit ED for psychiatric requirements are not considered as emergencies. This imposes a strain on resources that are required for true emergencies. The reasons for this include a lack of understanding on the patient's part and a lack of control on service providers. To address this issue, local campaigns to educate patients on where to seek help in mental health crises should be implemented. Similar campaigns against depression and suicidal tendencies were implemented in a major German city 20 years ago [30]. Concerning service providers, strategies include setting up a crisis hotline or opening crisis consultation hours within outpatient settings. These measures have been recommended by the German Council of Experts on the Improvement of Emergency Care [31]. In future studies, to reduce the burden on EDs, it is important to evaluate the motivations of patients with low urgency for seeking psychiatric care at EDs. A limitation of the study was the retrospective design. Due to this design nature, it is possible that patients who had a psychiatric condition, though were initially diagnosed with a physical ailment, were not included in this study. It must also be considered that the quality and reliability of secondary data can vary significantly. Since such data were collected for a different purpose, they might not have aligned perfectly with this study’s research objectives, leading to potential biases, errors, or missing information. The study only considered patients who visited the examined hospital and did not consider any previous treatment received elsewhere, as the study design could not exclude it. There is also a lack of data (socio-economic characteristics) due to the study design, rendering the interpretation of results and consequent conclusions considerably more challenging. Furthermore, only one hospital (across a 12-month period) was studied. The results obtained are specific to the investigated ED and cannot be generalized nationwide.

## Conclusions

Nearly one-third of patients did not have any emergency characteristics in this study, and consequently, proper help in a primary care setting would be appropriate. Only 10% of nonurgent patients had previous contact with a GP. The absence of timely and easily accessible outpatient therapy or care, as often experienced by patients, should be also considered as a potential cause of increased ED usage. First-time patients are more likely to not meet the criteria of a PE in comparison to patients with an existing history of previous consultations. Further research is required to understand more comprehensively the individual motivations behind this behaviour, or the possible failing of the healthcare system through failing to fulfilling its healthcare-providing mandate could be at stake. There could be both pull and push factors that play a role in this potential ED mismanagement, similar to the factors found in studies of somatic patients.

## Data Availability

The data presented in this study are available on request from the corresponding author. The data are not publicly available due to reasons pertaining to patient privacy.

## References

[1] Larkin GL, Claassen CA, Emond J (2005). Trends in U.S. emergency department visits for mental health conditions, 1992 to 2001. Psychiatric Serv.

[2] Hazlett SB (2004). Epidemiology of adult psychiatric visits to U.S. emergency departments. Acad Emerg Med.

[3] Capp R, Hardy R, Lindrooth R (2016). National trends in emergency department visits by adults with mental health disorders. J Emerg Med.

[4] Hooker EA, Mallow PJ, Oglesby MM (2019). Characteristics and trends of emergency department visits in the United States (2010–2014). J Emerg Med.

[5] Statistisches Bundesamt. 9,8 Millionen Behandlungen in Notfallambulanzen im Jahr 2021 (20.12.2022). https://www.destatis.de/DE/Presse/Pressemitteilungen/Zahl-der-Woche/2022/PD22_51_p002.html. Accessed 17 Sept 2023.

[6] Figura A, Kuhlmann SL, Rose M (2021). Mental health conditions in older multimorbid patients presenting to the emergency department for acute cardiac symptoms: cross-sectional findings from the EMASPOT study. Acad Emerg Med Off J Soc Acad Emerg Med.

[7] Duggan M, Harris B, Wai-Kwan C, Calder R. Nowhere else to go: why Australia’s health system results in people with mental illness getting ‘stuck’ in emergency departments. Mitchell Institute; Victoria University September 2020.

[8] Deutscher Ärzteverlag GmbH, Redaktion Deutsches Ärzteblatt. Zustände in den Notaufnahmen sind “erbärmlich” (27.04.2022). https://www.aerzteblatt.de/nachrichten/106908/Zustaende-in-den-Notaufnahmen-sind-erbaermlich. Accessed 17 Sept 2023.

[9] Sprivulis PC, Da Silva J-A, Jacobs IG (2006). The association between hospital overcrowding and mortality among patients admitted via Western Australian emergency departments. Med J Aust.

[10] Schlump C, Thom J, Boender TS (2022). Nutzung von Routinedaten aus Notaufnahmen zur Surveillance von Suizidversuchen und psychiatrischen Notfällen. Bundesgesundheitsbl.

[11] Freudenmann RW, Espe J, Lang D (2017). Psychiatrische Notfälle auf der medizinischen Notaufnahme des Universitätsklinikums Ulm in den Jahren 2000 und 2010. Psychiatr Prax.

[12] Clarke DE, Boyce-Gaudreau K, Sanderson A (2015). ED triage decision-making with mental health presentations: a "think aloud" study. J Emerg Nurs.

[13] Smart D, Pollard C, Walpole B (1999). Mental health triage in emergency medicine. Aust N Z J Psychiatry.

[14] Broadbent M, Moxham L, Dwyer T (2014). Implications of the emergency department triage environment on triage practice for clients with a mental illness at triage in an Australian context. Australasian Emerg Nurs J: AENJ.

[15] Goldman-Mellor S, Jia Y, Kwan K (2018). Syndromic surveillance of mental and substance use disorders: a validation study using emergency department chief complaints. Psychiatric Serv (Washington, D.C.).

[16] Thom J, Mauz E, Peitz D, Kersjes C, Aichberger M, Baumister H, Bramesfeld A, Daszkowski J, Eichhorn T, Gaebel W, Härter M, Jacobi F, Kuhn J, Lindert J, Markgraf J, Melchior H, Meyer-Lindenberg A, Nebe A, Orpana H, Peth J, Reininghaus U, Riedel-Heller S, Rose U, Schomerus G, Schuler D, Rüden U von, Hölling H. Aufbau einer Mental health surveillance in Deutschland: Entwicklung von Rahmenkonzept und Indikatorenset: Robert Koch-Institut; 2021.

[17] Reeves WC, Strine TW, Pratt LA, Thompson W, Ahluwalia I, Dhingra SS, McKnight-Eily LR, Harrison L, D’Angelo DV, Williams L, Morrow B, Gould D, Safran MA. Mental Illness Surveillance Among Adults in the United States. Morbid Mortal Wkly Rep. 2011;60:1–30. Centers for disease control and prevention.

[18] Wagner B, Diercke M, Kocher T et al. Syndromic surveillance of mental and substance use disorders: a validation study using emergency department chief complaints. Psychiatric Serv. 10.1176/appi.ps.201700028 28945179

[19] Schmiedhofer MH, Searle J, Slagman A (2017). Inanspruchnahme zentraler Notaufnahmen: Qualitative Erhebung der Motivation von Patientinnen und Patienten mit nichtdringlichem Behandlungsbedarf. Gesundheitswesen (Bundesverband der Arzte des Offentlichen Gesundheitsdienstes (Germany).

[20] Somasundaram R, Geissler A, Leidel BA (2018). Beweggründe für die Inanspruchnahme von Notaufnahmen – Ergebnisse einer Patientenbefragung. Gesundheitswesen Bundesverband der Arzte des Offentlichen Gesundheitsdienstes (Germany).

[21] Schmiedhofer M, Möckel M, Slagman M, Frick J, Ruhla S, Searle J. Patient motives behind low-acuity visits to the emergency department in Germany: a qualitative study comparing urban and rural sites. https://scholar.google.de/scholar?hl=de&as_sdt=0%2C5&q=schmiedhofer+2016+low-acuity&btnG=&oq=schmiedhofer+2016. Accessed 17 Sept 2023. 10.1136/bmjopen-2016-013323 PMC5129074 27852722

[22] Schmiedhofer M, Searle J, Slagman A et al. Perception of the emergency department for outpatient care in a rural region in Saxony-Anhalt: a qualitative survey of patients and general practitioners. Dtsch Med Wochenschr. 2017;142(10):e61–73. 10.1055/s-0043-100639 28355651

[23] Termin beim Psychotherapeuten: Kassenpatienten warten und warten und warten…: https://www.stern.de/gesundheit/termin-beim-psychotherapeuten--kassenpatienten-warten-im-schnitt-fuenf-monate-7937794.html. Accessed 17 Sept 2023.

[24] Hulsmans N, Sinani G, Ullrich H (2023). Vorstellung psychiatrischer Notfallpatienten in der Notaufnahme durch Notarzt und Rettungsdienst – Charakterisierung. Versorgung und Verbleib Notfall Rettungsmed.

[25] Rheinhold A, Greiner F, Schirrmeister W, Walcher F. Der Notfall „geht“ ins Krankenhaus. Eine Befragung von Patienten mit niedriger Dringlichkeit in einer Notfallaufnahme mit regionaler Alleinstellung. https://www.researchgate.net/publication/340640156_Der_Notfall_geht_ins_Krankenhaus_Eine_Befragung_von_Patienten_mit_niedriger_Dringlichkeit_in_einer_Notfallaufnahme_mit_regionaler_Alleinstellung. Accessed 17 Sept 2023. 10.1007/s00063-020-00681-4 32291507

[26] Reins L-M (2021). Analyse der Daten von Patienten der zentralen Notaufnahme am Universitätsklinikum Augsburg im Jahr 2017 [Dissertation].

[27] Reeves WC, Strine Tara W., Pratt LA, Thompson W, Ahluwalia IB, Dhingra SS, McKnight-Eily LR, Harrison L, D'Angelo DV, Williams L, Morrow B, Gould D, Safran MA. Mental illness surveillance among adults in the United States; 2011. 21881550

[28] Marchesi C, Brusamonti E, Borghi C (2004). Anxiety and depressive disorders in an emergency department ward of a general hospital: a control study. Emerg Med J: EMJ.

[29] Biancosino B, Vanni A, Marmai L (2009). Factors related to admission of psychiatric patients to medical wards from the general hospital emergency department: a 3-year study of urgent psychiatric consultations. Int J Psychiatry Med.

[30] Deutscher Ärzteverlag GmbH, Redaktion Deutsches Ärzteblatt. Optimierte Versorgung depressiver Patienten und Suizidprävention: Ergebnisse des „Nürnberger Bündnisses gegen Depression“ (22.05.2023). https://www.aerzteblatt.de/archiv/39338/Optimierte-Versorgung-depressiver-Patienten-und-Suizidpraevention-Ergebnisse-des-Nuernberger-Buendnisses-gegen-Depression. Accessed 17 Sept 2023.

[31] Regierungskommission für eine moderne und bedarfsgerechte Krankenhausversorgung. Reform der Notfall- und Akutversorgung in Deutschland. Integrierte Notfallzentren und Integrierte Leitstellen Februar 2023. https://www.bundesgesundheitsministerium.de/fileadmin/Dateien/3_Downloads/K/Krankenhausreform/Vierte_Stellungnahme_Regierungskommission_Notfall_ILS_und_INZ.pdf. Accessed 22 May 2023.

